# Uncomplicated circulatory shock: a narrative review

**DOI:** 10.31744/einstein_journal/2024RW0775

**Published:** 2024-10-21

**Authors:** Mauro Dirlando Conte de Oliveira, Oscar Fernando Pavão dos Santos, Giancarlo Colombo, Thiago Domingos Corrêa, Miguel Cendoroglo

**Affiliations:** 1 Hospital Israelita Albert Einstein São Paulo SP Brazil Hospital Israelita Albert Einstein, São Paulo, SP, Brazil.

**Keywords:** Shock, Hemodynamic monitoring, Fluid therapy, Cardiac output, Hemodynamics, Pulmonary embolism, Cardiac tamponade, Shock, cardiogenic, Shock, hemorrhagic, Anaphylaxis, Adrenal insufficiency, Shock, septic, Intensive care units

## Abstract

Circulatory shock is a common fatal condition. Despite this, information on this syndrome in the current medical literature is fragmented and esoteric. Adherence to each basic element of care can have a profound impact on patient outcomes. Disturbances in pumping (cardiogenic), content/container relationship (hypovolemic and vasoplegic), or blockage in blood circulation (obstructive) can induce tissue hypoperfusion, causing hemodynamic shock. If not quickly reversed, hypoperfusion progresses to irreversible multi-organ failure. The course can be fatal even before reaching this stage in cases of obstructive and anaphylactic shock in which the therapeutic window may last for only a few minutes. Thus, it is essential to conduct a joint analysis of clinical data and routine diagnostic tests to infer the probable cause of shock and avoid delays in the diagnosis of diseases that can deteriorate quickly. Point-of-care ultrasonography and echocardiography are the most valuable non-invasive diagnostic tools. Although lactate-guided management has proven to be effective, the use of capillary refill time, other biochemical markers of perfusion, and preload-directed resuscitation have the potential to avoid volume overload and improve outcomes. Faster intravenous fluid infusion and early use of vasopressors have a strong rational appeal. However, in hemorrhagic shock, finding a balance between avoiding excessive crystalloid administration and maintaining adequate perfusion pressure until hemostasis is achieved remains challenging. This review provides an accessible description of bedside management of shock, including the treatment of the main causes. The most relevant information has been organized into tables for quick reference.

## INTRODUCTION

The shock state is characterized by circulatory failure, which induces insufficient oxygen delivery to meet tissue demands. This condition is frequent (affecting 1/3 of the patients admitted to intensive care units) and is associated with a high mortality rate (38.3% in a large series).^(1)^

In 1971, Weil et al.^(2)^ proposed the following pathophysiological classification of shock, which is still in use: cardiogenic ("the cardiac pump is impaired to the extent that it cannot competently circulate available volume"), hypovolemic ("the volume contained within the intravascular compartment is inadequate for perfusion"), obstructive (physical blockage to the inflow or outflow of blood in the heart), and vasoplegic or distributive (peripheral circuit failure). This review explains shock in a practical and accessible manner.

## CLINICAL MANIFESTATIONS OF THE STATE OF CIRCULATORY SHOCK

Shocks can be divided into three phases: pre-shock, shock, and organ injury.^(3)^

### Pre-shock phase (compensated shock)

For practical reasons, pre-shock is considered the phase of tissue hypoperfusion that precedes the development of hypotension. Mean arterial pressure is the product of the cardiac output (CO) and systemic vascular resistance (SVR) added to the central venous pressure (CVP) (MAP = [CO × SVR] + CVP). Tissue hypoxemia induces sympathetic activation, which causes an increase in the CO (in vasoplegic shock) or peripheral vasoconstriction with an elevation in SVR (in other types of shock). Until exhaustion, these mechanisms maintain the pressure in the normal range but often fail to prevent hypoperfusion.

Since cutaneous circulation is devoid of self-regulation, in shock states this territory is at the mercy of the neurohumoral response. The skin becomes cold, pale, moist, and mottled as the capillary refill time (CRT) increases. Hyperlactatemia may occur at this stage.

### Shock phase

Shock phase can be identified based on clinical, hemodynamic, and biochemical findings. Clinically, the patient becomes hypotensive, while the evidence of hypoperfusion described for the pre-shock period becomes more pronounced. In addition, patients may experience oliguria (diuresis <0.5 mL/kg/h) and changes in consciousness and cognition. The hemodynamic patterns provided by the Swan-Ganz catheter for each pathophysiological type of shock are presented in Table 1.^(4)^

**Table 1:** Hemodynamic profile of shock

Type of shock		Preload	Contractility	After load	Tissue perfusion
		PCWP	CO	SVR	SvO_2_ %
Hypovolemic		↔ (early) or ↓ (late)	↔ (early) or ↓ (late)	↑	>65 (early) or <65 (late)
Cardiogenic*					
	LV failure	↑	↓	↑	<65
	RV dysfunction and failure	↔ or ↓	↔ (early) or ↓ (advanced)	↔ or ↑	<65
Distributive (vasoplegic)		↔ (early) or ↓ (late)	↑ (↓ sometimes)	↓	>65
Obstructive					
	Pulmonary thromboembolism, pneumothorax	↔ (early) or ↓ (late)	↔ (early) or ↓ (late)	↑	>65
	Cardiac tamponade	↑	↓	↑	<65

CO: cardiac output; LV: left ventricle; PCWP: pulmonary capillary wedge pressure; RV: right ventricle; SvO_2_: mixed venous oxygen saturation; SVR: systemic vascular resistance.

Hyperlactatemia and other biochemical consequences of shock on non-invasive monitoring are discussed below.

### Organ injury phase

Extended hypoperfusion induces cell damage and organ failure. In 2001, a controlled study showed that early resuscitation guided by pre-established hemodynamic goals reduced mortality in shock.^(5)^ Subsequent controlled studies reinforced the notion that early reversal of shock, rather than the use of invasive hemodynamic goals, was the cause of better outcomes.^(6-8)^

Once organ failure occurs, increasing the oxygen supply above normal levels has not been effective in improving prognosis.^(9)^

## CAUSES AND DIFFERENTIAL DIAGNOSIS

History taking and physical examination may indicate a diagnosis. Jugular stasis suggests cardiogenic or obstructive shock. Pulse asymmetry can be caused by aortic dissection.

All patients must undergo chest radiography, echocardiography, and electrocardiography. Blood tests include blood count; troponin, brain natriuretic peptide, D-dimer, arterial or venous blood gases, and lactate levels; coagulogram; and kidney and liver function tests.

In a study, of the 118 patients admitted in the emergency department, without an obvious source of hypotension, a multiorgan point-of-care ultrasonography shock protocol led to a change in the diagnostic hypothesis or treatment plan in ¼ of the cases.^(10)^ However, a randomized controlled trial of 273 patients with true undifferentiated shock showed an overall diagnostic accuracy similar to that of the standard of care (93.7% *versus* 93.6%).^(11)^ This study ended prior to the recruitment of the initially planned 400 patients. However, the usefulness of this technology during shock still needs to be determined.

Table 2 provides an overview of the main clinical and laboratory findings that may indicate the etiologyof shock.^(3,12-14)^

**Table 2:** Differential diagnosis of hemodynamic shock

Type	Etiology	History and physical examination	Jugular stasis (CVP, mmHg)	ScvO_2_ %	Electrocardiogram	Echocardiogram
Vasoplegic (66%)	Septic (64%)	Recent fever or signs of infection	Absent (<8)	>70	Not characteristic	Small heart chambers, normal or increased contractility, and inspiratory collapseof the inferior vena cava
	Anaphylactic	± Skin or mucosal eruptions, respiratory and/or gastrointestinal symptoms				
	Non septic SIRS	SIRS criteria + pancreatitis, trauma, or burn				
	Neurogenic	Traumatic brain injury or spinal cord injury				
	Adrenal crisis	Mental alteration, hypoglycemia, hyponatremia, hyperkalemia, fever, and hyperpigmentation			± increased amplitude of the T wave	
Cardiogenic (17%)	Cardiomyopathy-related	Chest pain (in left or right ventricular infarction) and dyspnoea (in heart failure)	Present (>12)	<70	ST elevation, left bundle branch block, and repolarization disorder	Segmental hypokinesia and reduced contractility
	Mechanical	Signs of aortic or mitral insufficiency and stenosis; rupture of the papillary musculature, chordae tendineae or septum			Left ventricular overload and repolarization disorders; left atrial overload	Suggestive findings
	Arrhythmic	Extreme tachyarrhythmias or bradyarrhythmias	Variable		Sets the diagnosis	
Hypovolemic (16%)	Hemorrhagic	Trauma or gastrointestinal bleeding	Absent (<8)	>70 or <70	Not characteristic	Small heart chambers, normal or increased contractility, and inspiratory collapse of the inferior vena cava
	Dehydration	Gastrointestinal loss, salt-wasting nephropathy, and losses to the third space (pancreatitis and intestinal obstruction)				
Obstructive (2%)	Massive pulmonary thromboembolism (increased ventricular afterload)	Conditions predisposing to embolism (immobilization or surgery < 30 days, cancer, and history of prior thromboembolism)	Present (>12)	<70 (in general)	T-wave inversion in the right precordials and S1Q3T3 pattern are consistent	Dilated right ventricle with reduced contractility
	Hypertensive pneumothorax (obstruction of venous return)	Decreased lung expansion, reduced breath sounds, and hyper-resonance to percussion over pneumothorax			Right or left axis deviation (15%)	Suggestive findings
	Cardiac tamponade (right ventricle diastolic filling reduction)	History of breast or lung cancer, kidney disease, hypothyroidism, or recent myocardial infarction; muffled noises; paradoxical pulse*; and/or Kussmaul's sign			Low voltage in precordial leads (69%), PR depression (12%), and electrical alternation^#^ (5%)	Systolic collapse of the right atrium and/or diastolic collapse of the right ventricle, and turgid inferior vena cava

* Reduction in SBP >10mmHg during spontaneous inspiration, mainly due to the inspiratory increase in systemic venous return in the right chambers, which induces septal displacement with reduced volume in the left chambers^#^ Alternation of QRS amplitude with each beat in one or all leads, without evidence of alteration in conduction pathways.

CVP: central venous pressure; S_cv_O_2_: central venous oxygen saturation; SIRS: systemic inflammatory response syndrome.

## HEMODYNAMIC MONITORING

Tissue perfusion can be assessed using both invasive and non-invasive methods.

### Non-invasive monitoring

#### Peripheral perfusion

Hallux temperature (ventral side) <27 °C correlates with a cardiac index <2 L/min/m^2^.^(15)^ In 60 patients with septic shock, the extent of mottled skin around the knee 6 h after resuscitation was better associated with mortality than serum lactate level, while MAP, CVP, and cardiac index showed no correlation.^(16)^ Capillary refill time was the first perfusion marker (peripheral or global) to normalize (≤3 s) after resuscitation (6 h).^(17)^ In another study, 416 patients with septic shock were randomized to continue fluid-responsive resuscitation until the CRT or lactate level was normalized or decreased to <20%. The 28-day mortality was 34.9% in the CRT group and 43.4% in the lactate group (hazard ratio, 0.75; 95% confidence interval [95%CI], 0.55-1.02], p=0.06).^(18)^A Bayesian reanalysis suggested that CRT-guided resuscitation may result in a lower mortality rate and faster resolution of organ dysfunction.^(19)^

#### Biochemical markers of global perfusion

##### Central venous oxygen saturation, S_cv_O_2_

S_cv_O_2_ <70% (normal value, 73-82%) indicates that hemoglobin (Hb) underwent greater extraction of O_2_ by cells to compensate for the reduction in tissue blood flow. However, in the early phase of septic shock or in some patients with septic shock who have already been resuscitated, due to impaired O_2_ extraction, S_cv_O_2_ is often >70%, even in the presence of an O_2_ debt.^(20)^

##### Venoarterial carbon dioxide tension gradient, P(cv - a)CO_2_

The P(cv - a)CO_2_ (normal value, 2-5 mmHg) better reflects the adequacy of CO to wash out accumulated carbon dioxide (CO_2_) than the presence of tissue hypoxemia. When the capillary flow is reduced, the CO_2_ produced is dissolved in a smaller volume of venous blood, and this marker increases to >6 mmHg, indicating that venous return is inadequate for washing CO_2_ from peripheral tissues. Thus, normal P(cv - a)CO_2_ levels do not necessarily exclude the possibility of tissue hypoxia.^(21)^

##### Ratio between the venoarterial carbon dioxide tension gradient and arteriovenous O_2_ content gradient, P(cv-a)CO_2_/C(a-cv)O_2_

The P(cv-a)CO_2_/C(a-cv)O_2_ expresses the ratio between O_2_ consumption and CO_2_ production (normal value, 0.67-1.30). CaO_2_ is the arterial oxygen concentration ([1.38 × Hb in g/dL × SaO_2_ in %] + [0.0031 × PaO_2_ in mmHg]); CcvO_2_ is the central venous oxygen concentration ([1.38 × Hb in g/dL × S_cv_O_2_ in %] + [0.0031 × PcvO_2_ in mmHg]). Tissue hypoperfusion causes a reduction in both, but the concentration of CO_2_ is less reduced, due to its anaerobic production, which increases P(cv-a)CO_2_/C(a-cv)O_2_ to values >1.4. It is one of the best markers of anaerobic metabolism, and one of its advantages over lactate is its rapid response to hemodynamic improvement.^(20)^

##### Serum lactate

When tissue O_2_ drops to critical levels, glucose metabolism generates lactate (normal value, 0.5-1 mmol/L), raising its serum level to >2 mmol/L. Hyperlactatemia should be considered as an indicator of hypoperfusion in patients with shock. However, these patients often maintain high lactate levels and do not respond to an increased oxygen supply because of aerobic glycolysis related to adrenergic stress, reduced hepatic lactate clearance, and/or mitochondrial dysfunction, which limits the pyruvate metabolism.^(22)^Thus, lactate monitoring combined with the use of P(cv-a)CO_2_/C(a-cv)O_2_ is strongly recommended to avoid excessive fluid resuscitation and inotrope use.

#### Invasive monitoring

##### Indications and types of devices

The ProCESS, ARISE, and ProMISe studies failed to demonstrate that treating septic shock based on invasive hemodynamic goals was more effective than conventional treatments.^(6-8)^ Thus, indications for invasive monitoring are speculative.

When MAP drops below 60 mmHg, the autoregulatory limit is exceeded and blood flow to the organs becomes pressure-dependent.^(23)^ The current guidelines recommend maintaining an MAP of approximately 65 mmHg.^(24)^

In critically ill patients, non-invasive and invasive systemic MAP measurements may differ by >20 mmHg, either higher or lower, in some cases.^(25)^Invasive monitoring is the gold standard, and non-invasive measurements may result in an actual MAP below the autoregulation range. Therefore, invasive pressure monitoring is prudent if there is a need to use vasopressors and if the signs (clinical or laboratory) of hypoperfusion persist despite achieving the target MAP.

Based on the principle that the amplitude of the systolic part of the arterial curve and pulse pressure (difference between systolic and diastolic arterial pressure) are proportional to stroke volume (SV; volume of blood pumped out of the left ventricle during each contraction), the arterial catheter can be connected to devices that analyze these variables to estimate CO.

These devices can be "calibrated" by simultaneously measuring CO through transpulmonary thermodilution (PiCCO, VolumeView/EV1000) or lithium dilution (LiDCOplus monitor), which allows for reliable CO measurements in unstable patients.^(26)^

"Uncalibrated" devices (FloTrac/Vigileo, ProAQT/Pulsioflex, LiDCOrapid/pulseCO, and MostCare) derive CO from a pulse wave contour analysis based on a predefined pattern. Therefore, they are less accurate in detecting significant short-term changes that may occur in vasoplegic shock requiring vasopressors.^(26)^

Although a recent systematic review of over 1,300 patients suggested a low risk of peripheral infusion of vasopressor agents,^(27)^ a statement from the Intensive Care Society of 2023 recommends that in most circumstances, this should be done as a bridging measure until a central venous access device is available or used for a short term under specific circumstances.^(28)^

The central route can also be used to measure the CVP and S_cv_O_2_ (PreSep or CeVOX devices). This information can also help to identify the type of shock (Table 2).

The Swan-Ganz catheter, introduced through a central vein, measures the CO, pressure in the right atrium, pressure in the pulmonary artery (the blood collected in this route allows SvO_2_ measurement), and pulmonary capillary wedge pressure (pulmonary artery occlusion pressure [PAOP]), which is indicative of left atrial filling pressure. The catheter also provides data for calculating pulmonary and systemic vascular resistance. As shown in Table 2, these parameters allow identification of the pathophysiological type of shock. Monitoring using Swan-Ganz catheters has not been effective in improving shock outcomes.^(29)^ However, it can be useful if the echocardiogram is not diagnostic, in shock refractory to standard treatment, or in the presence of right ventricular failure or pulmonary hypertension, because no other monitoring measures the pressures in these territories.^(4,26)^

#### Monitoring the heart preload and response to fluids and inotropes

Preload is defined as the degree of stretching of the cardiac myocyte prior to ventricular contraction, which is directly related to SV, described by the Frank-Starling curve. Fluid responsiveness indicates an increase in SV >10% after volume infusion, which occurs in only 50% of patients with shock.^(30)^ Thus, it is necessary to differentiate between responders and non-responders accurately.

Fluid responsiveness does not imply that a patient requires fluids. Generally, in responders, infusion should be continued until tissue perfusion normalizes (lactate and/or P(cv-a)CO_2_/C(a-cv)O_2_). Non-responders should be managed with vasopressors or inotropes because there is much evidence that unnecessary or excessive fluid administration is associated with worse outcomes.^(31,32)^A recent multicenter randomized trial showed that fluid restriction in patients with sepsis did not reduce the 90-day mortality. However, the difference between the restrictive and liberal fluid strategy groups was only 2 L,^(33)^ possibly too small to alter the outcome and significantly less than the cumulative fluid balance of tens of liters practiced in the recent past.

Static tests for assessing fluid responsiveness are based on estimating the preload through CVP, PAOP, end-diastolic volume (by echocardiography), or end-diastolic pressure of the right and left ventricles. These isolated measures are poor indicators of the volume status because there is no absolute value that discriminates responders from non-responders.^(34)^Static variables are affected by the systolic or diastolic dysfunction of the ventricles, valvopathy, and pulmonary vascular diseases.

Dynamic tests assess the increase in SV (or its derived variables) induced by increased venous return and preload. Inotropes can increase the SV, which is associated with a reduction in CVP and PAOP and transform non-responders into fluid responders.^(35)^Thus, the volume response should be reassessed after changes in vasoactive drug use. The dynamic maneuvers used to assess the preload responsiveness are described below:

#### Stroke volume variation (SVV) and pulse pressure variations (PPV)

Stroke volume variation and PPV indicate the differences between the maximum (inspiratory) and minimum (expiratory) left ventricular systolic volumes and perfusion pressures, respectively. The magnitude of this variation indicates that SV is dependent on biventricular preload.^(36)^ Stroke volume variation and PPV maintain their performance at an ejection fraction of <0.35.^(37)^ Pulse pressure variations appears to be the most reliable variable.^(38)^

The variations associated with fluid responsiveness, accuracy, and limitations of the most commonly used dynamic tests are listed in Table 3.^(30, 39-42)^

**Table 3:** The most studied dynamic fluid responsiveness tests

Method	Limitations	Maneuver or clinical condition	Indicates fluidresponsiveness	Sensitivity, % (95% CI)	Specificity, % (95% CI)
Central venous pressure	Right ventricular infarction or failurePulmonary hypertensionconstrictive pericarditisHypertensive pneumothorax	Decreased CVP with spontaneous inspiration	↓ ≥1 mmHg	94 (NA)	81 (NA)
Pulse pressure variation	Spontaneous ventilationCardiac arrhythmiaAcute respiratory distress syndromeRight ventricle failure	Controlled ventilation, Vt ≥ 7 mL/kg PBW	∆ ≥11%	84 (75-90)	84 (77-90)
		Increase in PPV from Vt 6-8 mL/kg PBW	­↑ ≥3.5%	94 (NA)	100 (NA)
Variation in systolic volume	Spontaneous ventilation Cardiac arrhythmia	Controlled ventilation, Vt ≥ 7 mL/kg PBW	∆ ≥13%	79 (67-87)	84 (74-90)
	Acute respiratory distress syndromeRight ventricle failure	Increase in SV variation from Vt 6-8 mL/kg PBW	­↑ ≥2.5%	88 (NA)	100 (NA)
Respiratory variation of inferior vena cava diameter	Spontaneous ventilationCardiac arrhythmiaAcute respiratory distress syndrome	Controlled ventilation, Vt ≥ 8 mL/kg PBW	∆ ≥15%	77 (44-94)	85 (49-97)
Cardiac output/cardiac index	Right ventricular infarction or failureIntrathoracic or intra-abdominal pressure >12 mmHgCardiac tamponade or tension pneumothoraxAdrenergic stimulation (pain, cough, or discomfort)	Passive lower limb raising	↑ cardiac output ≥11%	88 (80-93)	92 (89-95)
	Not intubated Unable to stop spontaneous breathing for at least 15s	End-expiratory occlusion test	↑ cardiac index ≥5%	91 (72-99)	100 (72-100)

* If the result is negative in ventilation with low tidal volume, pulse pressure and stroke volume variations must be reassessed after a transient increase (1 min) in tidal volume to 8mL/kg. Absolute increases ≥3.5% in pulse pressure variation and ≥2.5% in stroke volume variation indicate fluid responsiveness.

∆: variation; CVP: central venous pressure; NA: not applicable; PBW: predicted body weight; SV: stroke volume; Vt: tidal volume; 95%CI: 95% confidence interval.

#### Fluid infusion test

The test is performed with the patient in the horizontal dorsal decubitus for 3 min. Cardiac output is then estimated. Infusion of 4 mL/kg crystalloid over 5 min reliably differentiates responders from non-responders.^(43)^ The maximum increase in CO should be assessed 1 min after the infusion is completed. The patient is considered fluid-responsive if there is a minimum increase of 10% in CO, which ensures that this change does not result from measurement variability.

To avoid unnecessary administration of fluids, most recent studies evaluated the infusion of only 100 mL of crystalloid for 60 s, with the best limit set at 5% increase in the systolic volume and the parameters derived from it.^(44)^ The mini-fluid challenge requires an accurate estimate of CO.

#### Passive leg raising

The passive leg raising maneuver moves part of the blood from the venous beds of the lower limbs and abdomen to the intrathoracic compartment, which is equivalent to an infusion test of 300 mL of saline solution^(45)^ but without the disadvantage of adding volume in the case of a patient not responsive to fluids.

To perform the maneuver, the patient must be kept in the supine position for 3 min, without intermittent pneumatic compression, with the trunk elevated at 45° (to increase the volume of recruited blood), and then the baseline hemodynamic measurement must be performed. Subsequently, the position should be changed to horizontal and both lower limbs should be elevated to a 45° angle. The hemodynamic measurement is repeated after 60 s in this position, when the maneuver effect is at its maximum. Ideally, the results of passive leg raising should be evaluated by direct and real-time measurements of CO, such as those derived from pulse-wave contours, even if obtained using uncalibrated systems.^(46)^ The hemodynamic effects of the maneuver can also be evaluated using echocardiography and esophageal Doppler imaging, which estimate systolic volume beat-by-beat.^(46)^

#### End-expiratory occlusion test

In mechanically ventilated patients, each air insufflation increases the positive intrathoracic pressure, which reduces venous return and, consequently, the preload. Temporarily interrupting the respiratory cycle at the end of expiration reverses this effect, and if it induces an increase in CO, it indicates the responsiveness of both ventricles to preload.^(47)^

The test requires the CO to be estimated precisely and changes to be detected within a few seconds, such as through the analysis of the pulse-wave contour in a calibrated system.

The maneuver must be performed with the patient sedated in the supine position and the trunk elevated at 30°. Baseline CO should also be measured. Then, using a ventilator-specific device, periodic insufflations are interrupted in the final phase of expiration for at least 12 s(12-30 s) to allow time for the increase in direct cardiac preload to be transmitted to the left side. The pressure curve of the device is closely observed to ensure that spontaneous ventilation does not occur, and the CO is measured in the last 5 s of the maneuver when the change is at its maximum. The percentage of change relative to the baseline measurement is calculated.

Several new methods (invasive and non-invasive) to assess fluid responsiveness have been published,^(46)^ the discussion of which is beyond the scope of this review.

## TREATMENT OF CIRCULATORY SHOCK

The ultimate goal of shock treatment is to normalize hypoperfusion.

### Initial resuscitation

This refers to the rapid increase in venous return, CO, and effective intravascular volume induced by passive leg raising, the initial bolus and/or drugs employed before the installation of monitoring, and determination of volume status and cardiac function, which will determine the need for additional fluid and/or drugs. The goal is to reach an MAP of approximately 65 mmHg, and in the case of chronic hypertension, higher.^(48)^ Prolonged hypotension may be associated with high mortality rates.^(49)^

Even under normal pressure, cold and humid skin, purplish spots, or reduced CRT are indications for initiating volume resuscitation in patients with circulatory dysfunction.

#### Early use of vasopressors

One-third of patients do not respond to initial fluid resuscitation.^(50)^ Especially due to the delay in reaching an adequate MAP with the isolated use of fluids, early initiation of vasopressors has strong rational appeal. In the CENSOR trial, a randomized, double-blind, placebo-controlled study of 310 patients, early administration of norepinephrine reversed shock more rapidly and, although not statistically significant, resulted in lower mortality (15.5% versus 21.9%; relative risk [RR], 0.79, 95%CI= 0.53-1.11).^(51)^

#### Type of resuscitation fluid

There are uncertainties regarding the best solution for plasma expansion during a shock. In a controlled study of patients requiring volume resuscitation for various causes (SAFE study), the use of 4% albumin did not improve the outcome compared to saline^(52)^ and, in patients with head trauma and Glasgow coma score ≤13, it was associated with higher mortality.^(53)^ Hyperoncotic starch solutions are associated with high rates of acute kidney failure and death.^(54)^ Due to its high plasma chloride concentration, saline can induce hyperchloremic metabolic acidosis if administered in large volumes, and animal studies have shown that saline causes intrarenal vasoconstriction and reduces the glomerular filtration rate.^(55)^ A recent meta-analysis (34,450 participants) with a low risk of bias has shown that balanced crystalloids vs. saline presented RRs of 0.96 (95%CI= 0.91-1.01), 0.96 (95%CI= 0.89-1.02), and 0.95 (95%CI= 0.81-1.11) for mortality, development of acute kidney injury, and the need for renal re[placement therapy, respectively.^(56)^ This indicates the possible beneficial effect of balanced crystalloids when used in high volumes.

### Hemodynamic and metabolic optimization

In this phase, fine adjustments are made based on hemodynamic and metabolic responses.^(57)^ Measures to optimize the perfusion of organs and tissues depend on the assessment of the volume and functional status of the cardiac chambers. Echocardiography can be used to assess cardiac function and fluid responsiveness after passive leg raising. Limited amounts of intravenous fluid should be administered, followed by careful assessment of the patient's clinical response (*e.g.*, with the dynamic fluid response tests detailed above).

It is likely that the fluid infusion rate affects the outcomes. A post-hoc analysis of a randomized controlled trial involving 10,520 critically ill patients showed that a faster infusion rate (999 mL/h compared with 333 mL/h) was associated with a lower odds ratio for mortality (0.72; 95%CI= 0.54-0.91; probability of benefit >0.99) in a subgroup that included patients with sepsis.^(58)^

In patients with an MAP of 60-65 mmHg (with or without vasopressors), who are not fluid-responsive but have persistent signs of hypoperfusion or myocardial dysfunction, an inotrope should be added.^(59)^

### Treatment of specific causes of shock

Treatment should be initiated as the etiological investigation continues. In particular, in patients with distended jugular veins, the possibility of obstructive shock must be considered initially because the therapeutic window may last only a few minutes.

The section below describes the most relevant studies, and Table 4^(60-62)^ condenses this evidence into an objective framework for treating the main types of shock. The recommended vasoactive drugs and the most important aspects of specific treatments for each type of shock are also described.

**Table 4:** Relevant aspects of shock treatment

Type	Etiology		Initial crystalloid resuscitation	Vasoactive drugs	Specific treatment and other measures
Obstructive	Massive pulmonary thromboembolism		500–1,000 mL	Norepinephrine + dobutamine	Systemic thrombolysis or catheter-guided thrombectomy
	Hypertensive pneumothorax		1,000–2,000 mL	Vasopressor + inotrope	Needle thoracostomy in the second intercostal space in the midclavicular line or in the fifth intercostal space in the anterior axillary line
	Cardiac tamponade		500 mL, if SBP <100 mmHg	Norepinephrine ± dobutamine	Percutaneous pericardiocentesis guided by echocardiography or surgical drainage
Cardiogenic	Cardiomyopathy		300 mL, in the absence of pulmonary congestion	Norepinephrine ± dobutamine	In acute myocardial infarction, early revascularization of the involved coronary artery and dual antiplatelet therapy
	Mechanical*				
		Aortic stenosis	Not indicated	Vasopressin ± dobutamine	Balloon aortic valvuloplasty can be considered as a bridge to definitive treatment
		Aortic insufficiency	Not indicated	Dopamine ± temporary pacemaker to maintain elevated heart rate	Surgical aortic valve replacement or transcatheter aortic valve replacement
		Mitral stenosis	Not indicated	Vasopressin ± amiodarone to reduce heart rate	Balloon mitral valvuloplasty has a success rate of 65–80%. In degenerative mitral stenosis with severe calcification, transcatheter aortic valve replacement may be an option
		Mitral insufficiency	Not indicated	Norepinephrine ± dobutamine ± intra-aortic balloon pump	Ischemic papillary muscle rupture can be treated during myocardial revascularization. MitraClip^®^ has good results in primary or secondary mitral regurgitation
Hypovolemic	Dehydration		30 mL/kg as soon as possible or until signs of hypoperfusion reverse (whichever occurs first)	Norepinephrine only if there is a risk of cardiac arrest	Treat the underlying cause and correct electrolyte disturbances
	Hemorrhagic		500 mL aliquots aiming to maintain an SBP of 100 mmHg#	Vasopressin or norepinephrine	Early transfusion of 1:1:1 blood products or whole blood;Tranexamic acid in <3 h
Vasoplegic	Septic		30 mL/kg as soon as possible or until signs of hypoperfusion reverse (whichever occurs first)	Norepinephrine; if insufficient response, associate vasopressin± dobutamine	Start antibiotic in <1 hEmpirical antifungal treatment, if there are risk factorsControl of the source of infection
	Anaphylactic		1,000 mL in 1 to 3 min	Epinephrine 1mg intramuscularly every 5 min&	Delay in epinephrine administration worsens the outcome
	Adrenal crisis		1,000 mL in <1 h	Norepinephrine	Hydrocortisone 100 mg intramuscularly or intravenously, followed by 200 mg over 24 h
	Neurogenic£		1,000–2,000 mL	Norepinephrine	Maintain an MAP ≥85 mmHg in the first 7 days

* According to van Diepen et al.^(60)^ and Akodad et al.;^(61)^

# While blood derivatives are made available;

& If hypotension persists after the second dose, start continuous epinephrine (1mg of epinephrine in 100mL of 0.9% sodium chloride, at a flow rate of 0.5–1mL/kg/h);

£ In addition to hypotension, it is characterized by bradycardia, which helps to differentiate this type of shock from hypovolemic shock. Pressure target and vasopressor of choice are based on class III evidence (Lee et al.^(62)^).

SBP: systolic blood pressure; MAP: mean arterial pressure.

#### Treatment of obstructive shock

Shock results from a reduced right or left ventricular preload or afterload. Although some patients are fluid-responsive, volume resuscitation is only intended to get time to treat the causative event.

##### Massive pulmonary thromboembolism

Fluids must be administered with extreme caution. An increased right ventricular preload can worsen the stretching of the cardiac wall and induce ischemia and/or deviation of the septum towards the left ventricle, with the potential to reduce its compliance and filling, thus inducing a reduction in CO. However, if the CVP is <10 mmHg, fluids may increase right ventricular preload and cardiac index.^(63)^ The drugs indicated in cases of persistent hypotension or hypoperfusion are norepinephrine, which increases biventricular systolic volume and coronary perfusion without changing pulmonary vascular resistance, and dobutamine, which also has an inotropic effect and reduces filling pressure.^(64)^

Patients with systolic blood pressure (SBP) of <90 mmHg for 15 min or more and without a high risk of bleeding should receive systemic thrombolytic treatment (through a peripheral vein).^(65)^ A catheter-guided percutaneous removal of the thrombus is indicated for patients at high risk of bleeding, with failure of systemic thrombolysis, or with severe shock that can cause death within hours (before systemic thrombolysis takes effect).^(65)^

##### Hypertensive pneumothorax

Tension pneumothorax usually evolves into respiratory or circulatory failure, depending on whether the patient is on non-assisted or assisted ventilation.^(66)^ In patients on mechanical ventilation, sudden onset of hypotension, desaturation, or increased CVP and/or peak inspiratory pressure should be an alert to the possibility of tension pneumothorax.

Fluid resuscitation is less effective and requires substantial infusion volume.^(67)^ Vasopressors and inotropes prevent cardiac arrests while preparing for chest decompression. However, these recommendations are only speculative.

As more than 50% of patients develop sudden or acute hypotension or cardiac arrest,^(66)^ a needle thoracostomy (in the second intercostal space in the midclavicular line or in the fifth intercostal space in the anterior axillary line^(68)^) should be performed in patients with a compatible picture without waiting for radiographic confirmation. If the condition does not improve, a second needle decompression procedure can be performed. When effective, decompression restores venous return, promoting minimal stabilization until thoracostomy is performed with a pigtail catheter (for example, ≤14 Fr) or with a tube (24 or 28 Fr).

##### Cardiac tamponade

The right and left sides of the heart compete for a fixed intracardiac blood volume when cardiac filling is limited by fluid accumulation under pressure in the pericardial sac.^(69)^ Spontaneous inspiration induces a reduction in pleural pressure, which increases blood return to the right heart (shifting the septum to the left heart) and increases compliance with the pooling of blood in the pulmonary venous system. Both mechanisms result in decreased left ventricular filling and, consequently, a reduction in inspiratory CO and a drop in SBP by >10 mmHg. This characterizes a paradoxical pulse, which occurs in 98% of patients and can be detected accurately and in real-time with invasive blood pressure monitoring.^(70)^

In a study of patients with cardiac tamponade confirmed by hemodynamic criteria, fluid responsivenessoccurred only in patients with SBP <100 mmHg, whereas the cardiac index may decrease in the absence of hypotension.^(71)^ An inotropic agent should be administered if signs of hypoperfusion persist.

Specific treatment for cardiac tamponade is performed by draining the pericardial fluid, preferably in unstable patients, using a percutaneous needle or catheter pericardiocentesis guided by echocardiography.^(72)^ Surgical drainage is indicated in the presence of intrapericardial bleeding secondary to trauma, aortic dissection, or rupture of the post-infarction ventricular free wall, and in purulent pericarditis.

#### Treatment of cardiogenic shock

Volume resuscitation in patients with cardiomyopathic shock remains controversial. However, in a prospective study, approximately 50% of patients responded to a challenge with 300 mL of crystalloid with a significant increase in CO.^(73)^ Thus, this could be an initial approach in 1/4-1/3 of the patients who do not have pulmonary congestion.^(74)^

In direct comparison, norepinephrine was associated with a lower risk of death than dopamine.^(75)^ This drug has also been associated with a five times lower mortality rate than epinephrine.^(76)^ Norepinephrine does not significantly increase the heart rate or myocardial oxygen consumption.^(77)^

If clinical signs of hypoperfusion persist, an inotrope, such as dobutamine, should be added^(78)^ until invasive monitoring or echocardiography shows CO or left ventricular contractile function. However, in acute myocardial infarction, owing to the potential increase in myocardial oxygen demand with the use of intravenous inotropes, these drugs should be used at the lowest possible dose or postponed until revascularization occurs.^(60)^ Volume resuscitation and vasoactive drug management for acute right ventricular infarction are similar to those recommended above.^(79)^

In acute myocardial infarction, early revascularization^(80)^ and revascularization limited to the involved vessels^(81)^ are well-established approaches, whereas the intra-aortic balloon pump has not been shown to be effective.^(82)^Patients without contraindications should receive dual-antiplatelet therapy.^(60)^

Approximately one-fifth of the patients with cardiogenic shock secondary to acute myocardial infarction develop clinical manifestations of systemic inflammatory response syndrome (fever and/or leukocytosis) after 2-4 days, possibly due to sepsis.^(83)^ These patients present with relatively lower SVR.

The treatment options for other types of cardiogenic shock are summarized in Table 4.

#### Treatment of hypovolemic shock

##### Hypovolemic shock due to dehydration

Dehydration is primarily treated using crystalloid infusion. Saline and balanced crystalloids exhibit similar plasma expander effects. If large volumes of fluid need to be infused repeatedly, balanced crystalloids are preferred,^(56)^ but they should be avoided in cases of hyponatremia.^(84)^

The initial resuscitation volume is not defined. Therefore, we recommend the administration of a crystalloid as soon as possible in the same amount as recommended in patients with septic shock (30 mL/kg). Vasopressors can worsen tissue perfusion and should only be used temporarily to avoid cardiac arrest while continuing volume replacement.

##### Hemorrhagic shock

Aggressive crystalloid resuscitation before the bleeding source is controlled may accelerate blood loss due to increased intravascular hydrostatic pressure and/or the dilution of clotting factors. However, a recent systematic review (1,157 patients) found only a non-statistically significant lower mortality rate in resuscitation with hypotension (21.5% *versus* 28.6%).^(85)^ Although the immediate greater risk is the worsening of bleeding, after minutes to a few hours, the concern becomes the deleterious effect of shock. Because definitive evidence is lacking, it is reasonable to assume that in the absence of severe traumatic brain injury, a target systolic blood pressure of 100 mmHg may be sufficient to prevent hypoperfusion without worsening bleeding.^(86)^ Early use of blood products improves patient outcomes.^(87)^

In a randomized trial, the administration of thawed plasma in a pre-hospital setting resulted in lower mortality than standard-care resuscitation.^(88)^ Transfusion of plasma, platelet concentrate, and red blood cells at a ratio of 1:1:1 reduced early deaths from exsanguination compared with a ratio of 1:1:2,^(89)^ whereas whole blood may be even more effective than using blood components in equal parts.^(90)^

Early antifibrinolytic treatment (<3 h after injury) with tranexamic acid reduces death due to bleeding by 20%.^(91)^ In three retrospective analyses, vasopressor use was associated with increased mortality.^(92-94)^ But a randomized, double-blind study showed that the early use of vasopressin in 78 patients was associated with lower fluid volume resuscitation and no significantly lower mortality (25% *versus* 13%; p=0.19) at 5 days compared with the Control Group.^(95)^ Temporary use of vasopressin in cases of severe shock may prevent cardiorespiratory arrest, restrict the use of crystalloids, and maintain appropriate systemic perfusion until surgical hemostasis is achieved.

#### Treatment of vasoplegic shock

##### Anaphylactic shock

Shock results from vasodilation and increased vascular permeability.^(96)^ The use of epinephrine is associated with a rapid and consistent reversal of the condition.^(97)^Intravenous administration induces several adverse effects;^(98)^ therefore, the intramuscular route is preferred, even in patients with established venous access.^(99)^ For adults, the recommended dose is 0.5 mg, which can be administered every 5 min.^(100)^ If shock persists after the second dose, adrenaline infusion should be started (1 mg adrenaline in 100 mL of 0.9% sodium chloride, with an initial flow of 0.5-1 mL/kg/h). If the patient's response is inadequate, a second vasopressor should be administered.^(101)^ Delayed administration of epinephrine is associated with severe conditions and potentially fatal outcomes.^(102)^ Concurrently with epinephrine, 1,000 mL of crystalloid (pressurized) should be administered over 1-3 min and repeated as needed.^(97)^

##### Adrenal crisis

Insufficient production of glucocorticoids reduces the synthesis of enzymes that convert norepinephrine to epinephrine, which may account for shock and hypoglycemia.^(103)^ The clinical presentation can be confused with that of septic shock. After the collection of blood for adrenocorticotropic hormone, cortisol, creatinine, urea, sodium, potassium, glucose, and infection screening, treatment should be started without waiting for diagnostic confirmation. Treatment is based on the use of glucocorticoids and volume replacement. Hydrocortisone is recommended at an empirical dose of 100 mg, intramuscularly or intravenously, followed by 200 mg over 24 h (continuously or at a dose of 50 mg every 6 h).^(104)^ Initial resuscitation should be performed with 1 L of 0.9% sodium chloride in the first hour, with additional replacement based on fluid responsiveness (usually 4-6 L in the first 24 h).^(104)^ If glucocorticoids are not administered, hypotension may not respond to fluid or vasopressor treatment.^(105)^ Glucocorticoid and crystalloid replacement induces elevation of blood pressure within 4-6 h.^(106)^ The empirical use of antibiotics should be considered, as approximately 40% of patients are later found to have an infection.^(107)^

##### Septic shock

According to the 2021 Surviving Sepsis Campaign, treatment for septic shock should include timely recognition, empiric intravenous antimicrobials (preferably within 1 h of recognition), source control, fluids, vasopressors, and additional therapies.^(24)^ The guideline recommends the administration of 30 mL/kgof crystalloid within the initial 3 h of resuscitation.^(24)^ A similar volume was used in the ProCESS, ARISE, and ProMISe studies before randomization.^(108)^ Due to the previously described association between persistent hypotension and worse clinical outcome, 3 h may be too long to complete the initial fluid resuscitation. Norepinephrine is the first-line vasopressor. In cases of inadequate MAP levels, it is advisable to add vasopressin instead of escalating the dose of norepinephrine, and if necessary, to introduce epinephrine as a third drug.^(24)^In patients with adequate volume status and arterial blood pressure but persistent hypoperfusion due to cardiac dysfunction, dobutamine should be added to norepinephrine, or both drugs can be replaced by epinephrine alone.^(24)^

Based on the weak recommendation strength and moderate-quality evidence, the aforementioned consensus recommends the use of intravenous corticosteroids in patients who require ongoing vasopressor therapy.^(24)^ However, a patient-level meta-analysis published in 2023 suggested that the use of hydrocortisone was associated with more vasopressor-free days but not with increased survival.^(109)^ The only two trials that investigated the combination of hydrocortisone and fludrocortisone (1,541 patients) demonstrated a 14% reduction in mortality (95%CI= 0.79-0.92).^(109)^ The benefit may be due to improvements induced by mineralocorticoids in response to vasopressors.^(110)^

## FINAL COMMENTS

Circulatory shock is associated with a mortality rate of approximately 40-50%.^(1,75)^ Even in cases of sepsis, which is the most common type of shock, consensus recommendations are often ignored.^(111)^ Adherence to each basic element of care, which varies according to the primary mechanism, can have a profound impact on outcomes.^(111)^ This review assembled the best available evidence to provide an accessible discussion on the main practical aspects of the management of circulatory shock.
